# Binge Drinking, Cannabis Co-Consumption and Academic Achievement in First Year University Students in Spain: Academic Adjustment as a Mediator

**DOI:** 10.3390/ijerph17020542

**Published:** 2020-01-15

**Authors:** María Fernanda Páramo, Fernando Cadaveira, Carolina Tinajero, María Soledad Rodríguez

**Affiliations:** 1 Department of Developmental and Educational Psychology, Faculty of Psychology, C/ Xosé María Suárez Núñez, s/n, Campus Vida, 15782 Santiago de Compostela, Spain; carolina.tinajero@usc.es; 2 Department of Clinical Psychology and Psychobiology, Faculty of Psychology, C/ Xosé María Suárez Núñez, s/n, Campus Vida, 15782 Santiago de Compostela, Spain; fernando.cadaveira@usc.es; 3 Department of Social, Basic Psychology and Methodology, Faculty of Psychology, C/ Xosé María Suárez Núñez, s/n, Campus Vida, 15782 Santiago de Compostela, Spain; msoledad.rodriguez@usc.es

**Keywords:** alcohol binge drinking, cannabis, academic achievement, adjustment, university students

## Abstract

Little is known about how binge drinking or the combination of binge drinking and cannabis consumption affect academic achievement in students during the transition to university, or about the mechanisms that mediate this relationship. The purpose of this study was to evaluate the association between this pattern of alcohol/cannabis consumption and academic achievement, considering academic adjustment as a possible mediator. A total of 258 Spanish, first-year university students (145 females and 113 males), enrolled in undergraduate degree courses, were categorized into three groups on the basis of their patterns of alcohol/cannabis consumption: control, binge drinkers and co-consumers. The findings showed a significant effect of the combined binge drinking/cannabis consumption, but not of binge drinking alone, upon academic achievement and academic adjustment. Grade point average (GPA) and academic adjustment were lower in the co-consumers than in the other groups. Regarding the mediation effect, 34.33% of the impact of combined alcohol/cannabis use on GPA was mediated by academic adjustment. The combined consumption of alcohol and cannabis led to difficulties in adaptation to academic life, which in turn contributed to poorer performance at university. The implications of the findings are discussed.

## 1. Introduction

How do binge drinking and the co-consumption of alcohol and cannabis affect academic achievement in students during their transition to university? There is strong evidence that the use of legal and illegal drugs in the university population in Western countries has increased substantially in the last decade [1]. Binge drinking (BD), now recognized as a common pattern of alcohol consumption, is characterized by the intermittent consumption of four or more drinks (females) or six or more drinks (males), on a single occasion, usually at weekends, leading to a blood alcohol concentration (BAC) of 0.08 g/dL, at least once in the last 30 days [2]. This pattern of consumption is prevalent in first year Spanish university students [3]. The most recent national survey on the use of drugs in Spain [4] reports that the prevalence of BD among the population aged between 14 and 18 years is 32.3%. 

Many binge drinkers also consume other drugs as well as alcohol [5,6,7], and the most common combination appears to be alcohol and cannabis [8,9,10,11,12,13]. In Spain, approximately four out of ten binge-drinkers aged between 14 and 18 years report having used cannabis during BD episodes [4]. Furthermore, simultaneous consumption has been associated with an increased frequency and quantity of alcohol use [13,14,15,16,17]. 

The prevalence of the consumption of both substances is enhanced in the university context, where a climate of acceptance has been generated among university students due to a perception of low risk, as well as pro-cannabis campaigns and permissive policies [18,19,20,21]. Dealing in cannabis is a criminal offence in Spain, but cultivation or possession of cannabis for personal use is allowed if the quantity does not exceed 100 g. The factors that favor consumption overshadow empirical findings demonstrating the significant negative consequences on health [22,23,24,25], psychological well-being [26] and academic integration [18,27,28,29,30].

The first-year at university is not a homogeneous experience [31,32], consisting of a multiplicity of widely varying cultural, institutional, individual and family experiences with a decisive effect on the expectations of emerging adults [33,34]. Together these experiences determine how successfully students cope with the transition to university. It is therefore essential to understand the developmental changes that occur in this new context to enable the identification of risk trajectories in students entering higher education [35,36,37,38]. Although students generally look forward to the transition to university, the experiences and challenges that they face can be overwhelming. Thus, many students view their current lives as characterized by freedom and multiple possibilities, and they experience states of moratorium and instability in an unknown and highly competitive environment [39]. During the transition to university, students also encounter people with different frames of reference, as well as facing separation from the family and spending a greater amount of time with their peers. Through these interactions, students construct perceptions of normative behaviors that can influence their individual actions [40]. 

Adjustment to academic, personal–emotional and social demands [41] makes the initial entry period one of the most difficult times in higher education [36,42,43,44], and can lead to high levels of anxiety, interpersonal conflicts, feelings of isolation and loneliness, poor adaptation, academic failure and attrition [45,46,47,48,49,50,51]. In addition, many students may consider this stage as a period of emptiness or waiting, during which taking risks is an escape route whereby they can avoid facing uncertainty [43,52]. Recent research has shown that the transition to university marks a period of increased vulnerability to undertaking risky consumption, with BD and cannabis consumption becoming some of the most influential risk factors during this period [3,13,16,22,27,29,30,37,53,54,55]. 

Many studies have focused on the biological, neurocognitive and social consequences of BD, cannabis use, or the co-consumption of both alcohol and cannabis [17,23,37,56,57,58,59,60,61]. However, very few studies have examined how the co-consumption of these drugs affects academic performance in first-year university students, possibly because the association is very complex, and thus difficult to analyze and understand [27,62]. 

Cross-sectional research [63,64,65,66,67,68,69,70,71,72,73] and longitudinal studies [27,28,29,30,74,75,76,77] have confirmed that BD and cannabis use, whether carried out simultaneously or separately, contribute to poor academic performance. Students who use these drugs, especially co-consumers, spend less time studying, miss more classes, are less academically motivated, attain lower GPAs, and also have an elevated risk of university attrition. Furthermore, neurocognitive studies [23,78,79] have shown that consumption of these drugs is associated with impairments in working memory, learning, attention, planning ability and information processing, functions that are necessary for academic performance. 

The impact of BD and cannabis consumption on academic performance can occur alone and in combination with other factors, such as missing classes, infrequent study, low socioeconomic status, low academic self-efficacy and depression [18,27,29,72,76,80,81]. However, there is a gap in the scientific literature about mediators such as academic adjustment, which has proven to be a key predictor of academic performance in first-year undergraduates, as it fosters the attainment of goals and contributes to viewing the university experience as positive [42,51,66,82,83,84]. 

Academic adjustment is a variable process that involves many other factors beyond outcome-based academic performance [41,48], such as the motivation to complete academic requirements, academic effort and satisfaction with the academic environment. 

**Figure 1:**
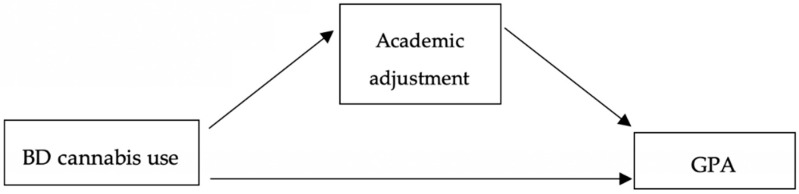
Mediation model for academic adjustment in the relationship between alcohol/cannabis consumption and grade point average (GPA).

This study aims to expand on previous research by evaluating the impact of BD alone, and also the BD-cannabis combination, on academic performance, considering academic adjustment, an unexplored phenomenon in first year Spanish university students, as a possible mediator. The hypotheses and conceptual model (Figure 1) of this study are as follows:

**Hypothesis** **1a** **(H1a).** *BD and co-consumption of alcohol and cannabis are associated with poor GPA.*

**Hypothesis** **1b** **(H1b).** *BD and co-consumption of alcohol and cannabis are associated with poor adjustment to university.*

**Hypothesis** **2** **(H2).** *Academic adjustment mediates the relationship between BD and cannabis use and GPA.*

## 2. Materials and Methods 

### 2.1. Participants and Procedure

The sample included 258 first-year students (145 females and 113 males), aged 18–19 years (M = 18.02, SD = 0.15), enrolled for the first time in undergraduate degree courses at the University of Santiago de Compostela (in Santiago de Compostela, Galicia, Spain). The majority of the students were single (91.7%), not working (97.7%), and came from families of middle socioeconomic status (85.3%).

The data used in the study were obtained in a large cross-sectional cohort study of university students enrolled in different degree programs. Participants were recruited through an anonymous survey that included demographic variables and items regarding the use of alcohol (AUDIT-C) [85,86], cannabis (CAST)) [87,88], tobacco [89,90] and other drugs. The following preselection criteria were applied to the completed classroom questionnaires: (1) provision of contact information (phone number, email) as a sign of willingness to enter subsequent phases of the study, (2) age, 18–19 years and (3) non-consumption of illegal drugs, except cannabis. The students thus selected were called for a clinical (face-to-face), structured interview. All participants provided written, informed consent, including consent to access data on their academic achievement. The study was approved by the Bioethics Committee of the University of Santiago de Compostela. 

Students noted their alcohol consumption habits in the six months prior to the interview (Alcohol Timeline Followback) [91] and their cannabis consumption in the three months prior to the interview. In order to examine the effects of alcohol and cannabis consumption on the outcome variables, we categorized participants into three groups on the basis of the number of BD episodes and scores for units of cannabis consumed (i.e., cannabis cigarettes or “joints”). Thus, students who had consumed four or more drinks (females) or six or more drinks (males) on a single occasion, at least once in the last 30 days, were classified as binge drinkers (BD). The BD-cannabis (BDCA) group consisted of BD students who had also consumed at least three cannabis units in the last 3 months. 

In order to collect a sample of “healthy” participants and reduce the possibility of other confounding variables, exclusion criteria for both groups were defined as follows: scores above 20 in AUDIT-C (cut-off point for possible abuse-related disorders or alcohol dependence); scoring in at least two symptomatic dimensions of the *Symptom Checklist*-*90*-R (SCL-90-R); uncorrected sensory deficits; personal history of traumatic brain injury or neurological disorder; personal history of any neurological or DSM-IV axis I disorder in first-degree relatives; and family history of alcoholism in first-degree relatives. 

### 2.2. Measures

Sociodemographic data on gender, mother´s and father´s educational level (primary school, high school, university), mother´s and father´s employment (unemployed, employed), socioeconomic status (low, middle, high) and the student’s place of residence (in family home, away from family home) were collected. The following variables related to substance use were considered: age of the onset of alcohol use, AUDIT-C scores, number of BD episodes (in the last 6 months), cannabis units consumed (in the last 3 months) and tobacco units consumed per day. 

#### 2.2.1. Adjustment to University 

The Spanish validated version of the Student Adaptation to College Questionnaire (SACQ) [41,92] was used to assess academic, social, personal–emotional and institutional adjustment of the students. The online version of the scale was administered to participants at the end of their first semester in their first year at university. This scale consists of 67 items scored on a 9-point scale ranging from 1 = *strongly disagree* to 9 = *strongly agree*. Negatively-worded items were reverse scored so that higher scores denoted better adjustment and composite scores were used for each following subscale. Academic adjustment (24 items, range 24–216) involves coping with the academic demands of the university experience. Social adjustment (20 items, range 20–180) assesses students’ feelings of fitting in, participating in social activities and making friends at university. Personal–emotional adjustment (15 items, range 15–135) focuses on the student’s psychological state and the extent to which he or she is experiencing general psychological distress. Institutional adjustment (15 items, range 15–135) assesses the feeling of attachment the students have to the particular institution they are attending and the quality of the relationship between the students and the institution. The Cronbach α was 0.90 for academic adjustment, 0.86 for social adjustment, 0.87 for personal–emotional adjustment, and 0.85 for institutional attachment.

#### 2.2.2. Academic Achievement

Academic achievement was assessed by the grade point average (GPA), obtained for each participant from the university’s central administration system at the end of first year. In the Spanish educational system, grades range from 1 to 10, and a mark of 5 or higher is required as a pass.

### 2.3. Statistical Analysis

Prior to the analysis, the data were screened for the presence of outliers, missing data and assumptions of normality and homogeneity of variance among groups. Descriptive characteristics of the study sample and each group were obtained for several baseline demographic variables (e.g., mother´s and father´s educational level) to isolate the effects of alcohol and cannabis use on outcomes (GPA and adjustment). The differences between groups in variables related to drug consumption (age of onset of alcohol use, number of BD episodes in the last 6 months, cannabis units in the last 3 months, tobacco units per day and AUDIT-C) were examined. Chi-square (*χ*^2^) tests were used to test differences in proportions, and one-way analysis of variance (ANOVA) and Scheffe post hoc-tests were used to compare means. 

One-way ANOVAs were used to assess mean differences in academic performance and the dimensions of adjustment to university (academic, social, personal–emotional adjustment and institutional attachment). Scheffe and Tukey post-hoc analysis was performed to identify which groups were different. Variables with *p* values ≤ 0.05 were considered statistically significant.

A multiple linear regression model was then computed to explore whether alcohol/cannabis use remained significantly associated with GPA after controlling for academic adjustment. Mediation analysis conducted with the PROCESS macro [93] estimated both the direct and indirect effects of alcohol/cannabis use on GPA, mediated by academic adjustment. A total of 5000 bootstrap samples were used to estimate the 95% confidence intervals (CIs), which indicate a significant effect if they do not include 0. In this model, the consumption group served as the independent variable, the average GPA served as the dependent variable, and academic adjustment was the mediator.

All analyses were conducted using the IBM Statistical Package (SPSS; Version 24.0, IBM Corp., Armonk, NY, USA).

## 3. Results

### 3.1. Socio-demographic Characteristics of the Study Participants 

Descriptive characteristics of the study sample (n = 258) are summarized in Table 1 for each group of participants: controls (n = 105), BDs (n = 89) and BDCAs (n = 64). The groups did not differ in gender, mother´s or father´s educational level, mother´s or father´s employment or socioeconomic status. However, there was an association between group and place of residence during term time (*χ*^2^ = 8.64, *p* = 0.1), with a higher proportion of BDCA participants living away from home (95.3%). Sociodemographic characteristics of the study participants were not significantly associated with the outcome variables.

Differences between groups in all variables related to substance use were detected. Post-hoc analysis showed that the number of BD episodes and AUDIT-C scores differed between all groups, and the mean scores were highest in the BDCA group. In addition, the mean age of the onset of alcohol use was lower in the BD and BDCA groups than in the controls. The data also showed that cannabis use increased with the number of BD episodes as well as with tobacco use. The BDCA students engaged in a greater number of BD episodes (M = 31.29, SD = 12.89), and consumed more units of tobacco per day (M = 1.18, SD = 2.34). The mean number of cannabis units consumed in the three prior months to evaluation was 19.12 ± 26.90 for the BDCA students.

### 3.2. Differences in GPA and Adjustment to University

Preliminary analysis conducted to examine the effect of gender and place of residence did not reveal any statistically significant differences or interaction effects (gender × group, place of residence × group) on academic adjustment and GPA.

**Figure 2:**
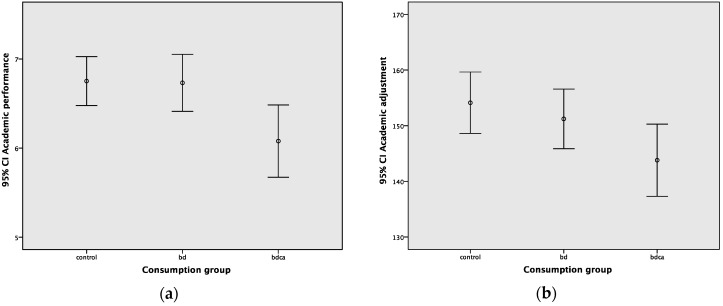
(**a**) Academic performance in the different groups; (**b**) Academic adjustment in the different groups. GPA and academic adjustment were lower in the BDCA group. BD: binge drinking. BDCA: binge drinking and co-consumption of cannabis.

Average academic performance and dimensions of adjustment to university are shown in Table 2. The mean GPA of the total sample was 6.58 ± 1.53. The differences in mean GPA between groups was statistically significant (F_2255_ = 4.69, *p* = 0.010, η^2^ = 0.036). Post-hoc analysis showed that the mean GPA was lower in the BDCA group (M = 6.08) than in the control group (M = 6.75) and the BD group (M = 6.73) (Figure 2a).

There were also significant differences in academic adjustment (F_2255_= 2.98, *p* = 0.045, η^2^ = 0.023). Academic adjustment was lower in the BDCA group (M = 143.80) than in the control group (M = 154.12), but there was no difference between the BD and BDCA groups (Figure 2b). There were no other differences in social, personal–emotional and institutional adjustment between these two groups.

### 3.3. Mediation Analysis

**Figure 3:**
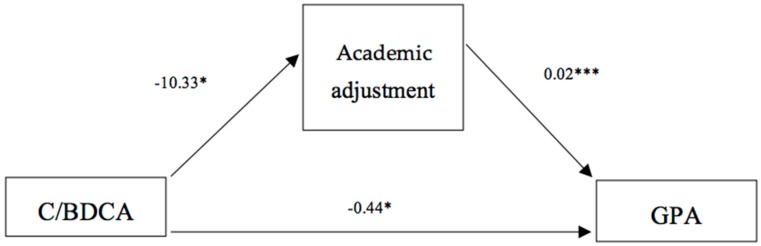
Mediation model tested. The relation between co-consumption and GPA was mediated by academic adjustment (* *p* < 0.05, *** *p* < 0.001). C/BDCA: Control vs. BDCA.

GPA and academic adjustment appear to be associated with BD and cannabis consumption. Direct and indirect effects were examined in order to assess whether academic adjustment is one of the mechanisms driving the effects of alcohol/cannabis consumption on achievement. Regression coefficients are presented as the unstandardized regression coefficients from the PROCESS macro (Figure 3). Mediation analysis showed that GPA was negatively influenced by the consumption of BDCA (relative to controls) and positively influenced by academic adjustment, which together explained 20.5% of the variance in GPA. Relative total, direct and indirect effects of alcohol/cannabis use on GPA were significant (Table 3). In addition to the direct relationship between alcohol/cannabis use and GPA (effect = −0.44; 95% CI = −0.88, −0.01), mediation analysis indicated that alcohol/cannabis use had an indirect effect on GPA (effect = −0.23; 95% CI = −0.48, −0.04), through its effect on academic adjustment. The ratio of indirect to total effect showed that 34.33% of the effect of alcohol/cannabis use on GPA was mediated by academic adjustment.

## 4. Discussion

The high drop-out rates and proportion of first-year students who change degree courses indicate that the transition to higher education is a critical period that should be analyzed with a view to possible intervention. The prevalence of a binge drinking pattern of alcohol consumption and co-consumption with cannabis, which reach maximal levels at this stage of life, may explain some of the difficulties associated with the transition to university. Previous data obtained by our research group [22] coincide with other studies in highlighting an increase in alcohol and cannabis use among first year university students in Spain [4,56]. The present study is one of the first of its type to assess academic performance and academic adjustment among controls, binge drinkers and alcohol and cannabis consumers in a cohort of first year university students in Spain. In addition, we tested whether academic adjustment mediates the effects of BD and cannabis consumption on academic achievement. On the basis of previously published information on the academic consequences of substance use among university students, we hypothesized that BD and the co-consumption of alcohol and cannabis will be associated with poor academic achievement. This hypothesis was partly confirmed, as the results revealed that GPA was lower in the BDCA group than in the control and BD groups, but that there was no difference between the last two groups. Although few studies have investigated the association between binge drinking and academic achievement in the university population, our study findings contradict some previous reports indicating heavy drinking and the frequency of alcohol use as negative predictors of the cumulative GPA [71,72,73,76,77]. However, our findings are consistent with longitudinal [94], cross-sectional [95,96] and prospective [55] studies reporting no effect, or a markedly attenuated effect, on academic performance among university students, after controlling for other predictors of academic failure (e.g., prior academic achievement, background variables, study habits, academic aptitude and cannabis use), or when including objective indicators of academic achievement [18,64]. Furthermore, our findings echo previous observations of an association between academic performance and alcohol dependence, but not alcohol abuse [63]. The evidence seems to indicate some weakness in academic achievement linked to BD. First, regarding problems related to the conceptualization of alcohol use, some studies examined binge drinking [64], while others examined consumption variables [55,71]. Second, different methods were used to measure the variables; thus, some studies [70,71,76,96] used objective measures (GPA), while others used subjective indicators [96]. Finally, when a number of plausible factors are controlled for, the effect of alcohol consumption upon academic performance may be negligible in healthy, young binge drinkers [94].

Thus far we have reported the effect of BD on academic achievement. Although the relationship between BD and academic achievement in university students has received some attention, the association between different types of consumption and academic performance in first-year university students has been less well studied. Only two studies have examined this association, in contrast to several studies that consistently provide evidence of academic problems associated with cannabis use in university students, such as lower GPA, lower exam and project grades, less time spent studying, decreased attendance and a higher drop-out rate [9,27,29,30]. In the present study, we expected academic performance to be poorer in the BDCA group than in the other groups. The findings show that co-consumption of alcohol and cannabis had a significant negative impact upon academic achievement. This finding was consistent with those of prior studies [18,29], which reported that the combined effect of both drugs is a significant predictor of lower GPA. Similar results have been obtained in studies of the co-consumers of tobacco, cannabis and alcohol which were studied in a university population [65] and a secondary school [28]. One potential explanation for these results is that co-consumption may be a greater risk factor for adverse academic outcomes than the risk posed by cannabis use alone [14]. Specifically, deficits in intellectual functioning, attention, memory, processing speed and visuospatial functioning were observed [14,24,61]. Such deficits can lead to lower academic motivation, poorer study skills, disrupted attendance and dropping out of university [27]. However, the body of research on the adverse consequences of the combination of binge drinking and cannabis consumption during university transition is surprisingly meager, given that peak consumption occurs at this stage.

Academic adjustment is considered a variable process that determines the subsequent path to higher education [41]. We hypothesized that BD and co-consumption of alcohol and cannabis will be associated with poor adjustment to university. We found that academic adjustment was significantly lower in the BDCA group. Again, our hypothesis was partly confirmed, as although academic adjustment was lower in binge drinkers than in controls, there was no difference between BD and BDCA groups (as determined by post-hoc analysis). One possible explanation is that drug use acts as a compensatory mechanism for coping with high levels of stress associated with the academic changes that occur during the transition from secondary to higher education, including adjustment to a highly competitive environment, the development of independent time management, new programs, new teaching practices and new institutional expectations [32,42,45]. Further research is needed to clarify the relationship between drug use and academic adjustment. 

In addition, the present study examined the mechanisms that might explain the association between the co-consumption of alcohol and other drugs and academic achievement. As mentioned above, the mediation role that academic adjustment plays in the association between BDCA and academic achievement is an unexplored phenomenon in first year university students in Spain and other countries. Specifically, we expected that frequent consumption of alcohol and cannabis would make it difficult for students to adapt adequately to academic life, which in turn would contribute to poorer performance at university. The study findings confirm the proposed model, i.e., that co-consumption contributes indirectly to academic outcome, with 34.33% of the effect of alcohol/cannabis use on academic performance being mediated by academic adjustment. In other words, alcohol and cannabis co-consumers perceived feelings of dissatisfaction and disengagement from university life, such as a lower motivation to complete academic requirements and lower academic effort, leading to academic failure. Despite the difficulties in explaining academic achievement in co-consumers on the basis of academic adjustment, many previous studies include academic adjustment as a powerful predictor of university outcomes [39,42,46,51,84], or as a mediator of the relationship between drinking motives and alcohol consequences [66]. 

The results of this research may be useful for clarifying the relationship between alcohol and cannabis use and academic performance, and for assessing the mediator role of academic adjustment in that relationship. Thus, although the possible association between BD and academic performance remains open, the poor performance in young co-consumers of alcohol and cannabis was confirmed. In addition, without underestimating the role that other factors may play, the important mediator role of academic adjustment was confirmed. Finally, it seems particularly relevant to us to note the role played by academic adjustment, as this leads the way to designing support programs in higher education institutions that specifically focus on risk groups, such as alcohol and cannabis co-consumers. Collaboration between health professionals, educational psychologists and academic institutions is essential for implementing intervention programs. 

Despite these important findings, this study has several limitations that should be considered. First, the sample was selected from one university, and should be extended to include other universities. Second, inclusion of a group of students who only consume cannabis would also represent an improvement, but this was not possible in the present study due to the low incidence of this type of drug consumption in the study sample. For reasons of experimental control, this preliminary study also did not include BDs with a family history of alcoholism or BDs with clinical-level problems, which could provide some information about the associated risk factors. In addition, application of these criteria excluded a large number of students at risk of academic failure. Third, as the study was cross-sectional, the association may not be consistent for any comparison of students in different years. Longitudinal studies would lend credibility to the relevant conclusions. Fourth, the study design did not consider the simultaneous use of cannabis and alcohol, or use within the same period. Consideration of both of these aspects would have improved the study and are of potential interest for future research. Fifth, the mediation model used did not allow us to reach any conclusions about the direction of academic adjustment. However, the cross-sectional study design does not allow the proposal of a causal order in the relationship between the variables studied. 

Finally, the weight of variables such as pre-university achievement [51,97], background variables [30], social support [98] and health problems [72] should also be tested in a more complete version of the model. 

## 5. Conclusions

This study provides some preliminary findings and contributes to addressing the lack of knowledge about the impact of binge drinking and of binge drinking in combination with cannabis consumption on academic performance in first year university students in Spain. Although no evidence was found for an association between binge drinking and either academic performance or academic adjustment, our findings highlight the presence of an association between the co-consumption of alcohol and cannabis and lower GPA and lower academic adjustment. A novel finding of this study is the mediating role of academic adjustment in this association. More specifically, we found support for our hypothesis that the relationship between the combination of binge drinking and cannabis use would be mediated by academic adjustment. The findings showed that the co-consumption pattern negatively impacts academic outcomes through poorer academic adjustment. These findings emphasize the importance of academic adjustment as a powerful predictor of academic performance. Thus, the study findings lay the foundations for designing support programs aimed at higher education and specifically focused on risk groups such as alcohol and cannabis co-consumers.

## Figures and Tables

**Table 1:** Characteristics of the overall sample and each consumer group [% or M (SD), and *p*-values for comparative statistics].

Variable	Total (n = 258)	Control (n = 105)	BD (n = 89)	BDCA (n = 64)	*p*-Value
Gender					
Male	43.8	46.7	40.4	43.8	0.68
Female	56.2	53.3	59.6	56.2	
Mother’s educational level					
Primary school	30.7	34.3	29.1	27.0	0.48
High school	24.4	20.0	30.2	23.8	
University	44.9	45.7	40.7	49.2	
Father’s educational level					
Primary school	36.7	35.3	41.7	32.3	0.55
High school	23.8	27.5	21.4	21.0	
University	39.5	37.3	36.9	46.8	
Mother´s employment					
Unemployed	23.8	29.7	19.0	20.6	0.19
Employed	76.2	70.3	81.0	79.4	
Father’s employment					
Unemployed	11.2	12.0	10.0	11.3	0.91
Employed	88.8	88.0	90.0	88.7	
Socioeconomic status					
Low	10.8	13.5	8.0	10.0	0.24
Medium	85.3	85.6	86.2	83.3	
High	4.0	1.0	5.7	6.7	
Residence					
In family home	14.3	21.0	13.5	4.7	0.01
Away from home	85.7	79.0	86.5	95.3	
Age of onset of alcohol use	15.70 (1.24)	16.36 (1.16)	15.46 (1.18)	15.21 (1.06)	<0.001
Number BD ^a^ episodes	15.33 (15.50)	1.15 (1.63)	20.57 (11.27)	31.29 (12.89)	<0.001
Cannabis units ^b^	4.81 (15.66)	0 (0)	0.19 (.56)	19.12 (26.90)	<0.001
Tobacco units day-1	0.45 (1.70)	0 (0)	0.51 (2.05)	1.18 (2.34)	<0.001
AUDIT-C	3.77 (2.56)	1.44 (1.46)	4.93 (1.89)	6.00 (1.52)	<0.001

^a^ In the six prior months to evaluation according to the Alcohol Timeline Followback [TLFB]. ^b^ In the three prior months to evaluation according to the record of cannabis consumption.

**Table 2:** Academic performance and adjustment in the overall sample and each group of participants. Means (M), standard deviations (SD) and *p*-value.

	Total (n = 258)	Control (n = 105)	BD (n = 89)	BDCA (n = 64)	*p*-Value
	M (SD)	M (SD)	M (SD)	M (SD)	
GPA	6.58 (1.53)	6.75 (1.42)	6.73 (1.52)	6.08 (1.62)	0.010
Academic adjustment	150.56 (27.05)	154.12 (28.50)	151.23 (25.41)	143.80 (25.99)	0.045
Social adjustment	138.43 (20.09)	136.93 (20.84)	138.77 (19.70)	140.43 (20.10)	0.538
Personal–emotional adjustment	91.67 (21.01)	91.46 (22.31)	92.46 (21.52)	90.94 (18.21)	0.899
Institutional attachment	112.60 (15.56)	112.40 (15.67)	112.56 (15.17)	112.99 (15.56)	0.972

**Table 3:** Relative total, direct and indirect effects of alcohol/cannabis use on GPA mediated by academic adjustment.

	Effect	Boot SE	Lower 95% CI	Upper 95% CI	*p* Value
Total effect	−0.67	0.24	−1.14	−0.20	0.005
Direct effect	−0.44	0.22	−0.88	−0.01	0.046
Indirect effect	−0.23	0.11	−0.48	−0.04	
